# The progesterone receptor PROGINS polymorphism is not related to oxidative stress factors in women with polycystic ovary syndrome

**DOI:** 10.1186/1475-2840-6-29

**Published:** 2007-10-05

**Authors:** Muammer Karadeniz, Mehmet Erdogan, Afig Berdeli, Sadik Tamsel, Fusun Saygili, Candeger Yilmaz

**Affiliations:** 1 Division of Internal Disease, Department of Endocrinology and Metabolism, Ege University, Izmir, 35100, Turkey; 2 Division of Molecular Medicine Laboratory, Department of Pediatrics, Ege University, Izmir, 35100, Turkey; 3 Division of Radiology, Ege University, Izmir, 35100, Turkey

## Abstract

**Background:**

Women with PCOS have been reported to be at increased risk of a number of gynaecological neoplasias, including endometrial, breast, and ovarian cancer. Studies of the possible association of genetic variation in progesterone receptor polymorphism with risk of ovarian and breast cancer have concentrated on a variant known as PROGINS.

**Methods:**

Ninety-five young women with PCOS and 99 healthy control women were included in our study. All subjects underwent venous blood drawing for complete hormonal assays, lipid profile, glucose, insulin and *PROGINS *polymorphism genetic study.

**Results:**

In PROGINS polymorphism results; in both control and the patient groups T1/T1 has been detected in high levels. But for genotype (p = 0.178) and allele (p = 0.555) frequencies both of the groups give similar results. Statistically significant difference has been detected on serum FSH levels for T1/T1 genotype according to T2/T2 genotype.

**Conclusion:**

No relation has been detected between the inflammatory and oxidative stress factors, and PROGINS polymorphism alleles. This may be because the PCOS patients are young and their BMI means are normal and their CIMT and oxidative stress markers are like healthy women.

## Background

Polycystic ovary syndrome (PCOS) is one of the most encountered endocrine malfunctions, which typically occur with chronic anovulation and hyperandrogenism [1]. Almost 15% of the women at reproductive period complain about PCOS [2]. Women with PCOS have been reported to be at increased risk of a number of gynaecological neoplasias, including endometrial, breast, and ovarian cancer [3]. The reproductive abnormalities in PCOS include chronic anovulation, prolonged exposure to oestrogen, progesterone deficiency, and androgen excess, which may contribute to an increased risk for gynaecological cancers in which the hormonal milieu is an important contributor to aetiology and prognosis.

Oxidative damage has been implicated in the pathogenesis of many chronic progressive diseases, such as cancer, inflammation, and neurodegenerative disorders. Emerging risk factors such as oxidative stress, inflammation and endothelial cell activation are thought to play integral roles in the development of atherosclerotic lesions and cancer [4,5].

In women with PCOS who are anovulatory or oligo-ovulatory, the regulatory roles of progesterone and progesterone withdrawal in the endometrial tissue are suboptimal or absent. In previously studies, heterozygous PROGINS constitution might lead to an over expression of progesterone inducible target genes even at physiologic levels of progesterone [6]. Moreover, this mechanism may contribute to the process of malignant transformation, atherosclerosis, Oxidative stress, insulin resistance.

The human progesterone receptor is a single copy gene located on chromosome 11q22-23 [7]. The actions of progesterone are mediated by two functionally distinct receptor isoforms, PR-A and PR-B, which are expressed from a single gene as a result of transcription from two alternative promoters [8]. Progesterone acts as a mitogen for breast tissue [9,10], and adding progestin's to menopausal oestrogen therapy increases mammographyical density, which is in turn associated with breast cancer risk [10]. Studies of the possible association of genetic variation in PGR with risk of ovarian [11-13] and breast cancer [14] have concentrated on a variant known as PROGINS. Progesterone is involved in the regulation of VSMC proliferation and modulates the synthesis of proinflammatory proteins such as monocyte chemoattractant protein-1, matrix metalloproteinase, E-selectin, and intercellular adhesion molecule-1 [15,16].

It is not known what impact PROGINS polymorphism may have on the inflammation process of the vasculature. The purpose of present study is to investigate the insulin resistance, inflammatory markers (hs-CRP, fibrinogen) and PROGINS Polymorphisms in young women with PCOS.

## Methods

Informed consent had been obtained before study in all patients and controls. Ninety-five young women (mean age, 24.27 ± 5.44 SD years) with PCOS and 99 healthy control women (mean age, 26.41 ± 5.65 SD years) were included in our study. PCOS was defined by the Rotterdam PCOS consensus criteria [17]. In all subjects (patients and controls) baseline plasma concentrations of LH, FSH, total testosterone, 17-hydroxyprogesterone (17-OHP), dihydroepiandrosterone sulphate (DHEA-S), oestradiol, SHBG and prolactin (PRL) were determined during the follicular phase (cycle days 5–7) after spontaneous or progestin-induced cycles.

Patients who had DM, hyperprolactinemia, congenital adrenal hyperplasia (diagnosed with the adrenocorticotropic hormone stimulation test), thyroid disorders, Cushing's syndrome, hypertension, hepatic or renal dysfunction were excluded from the study. Confounding medications, including oral contraceptive agents, hypertensive medications and insulin sensitizing drugs, and those, which may affect the metabolic criteria, were questioned.

Initially, ninety-nine patients were taken to the study but 4 patients were left voluntarily due to different personal reasons (*genotype success rate = 96,6%*). Similarly, 5 individual of 103 individuals were left the study voluntarily (*genotype success rate = 95,8%*). In all subjects, a clinical examination and an evaluation of hirsutism score by the Ferriman-Gallwey Classification were performed for each woman, a BMI (determined as weight in kg/height in m) were evaluated [18]. Individuals participating in the control group were the individuals who had laboratorial normal f-testosterone, total – testosterone, FSH, LH and basal insulin without acne, alopecia, menstrual disorder, and hirsutism. Healthy young volunteer females matched for age, body mass index (BMI), and allele frequency, were included and considered as the control group. Their healthy state was determined by medical history, physical and pelvic examination, and complete blood chemistry.

### Study protocol

At study entry, all subjects underwent venous blood drawing for complete hormonal assays, lipid profile, glucose, and insulin and *PROGINS *polymorphism genetic study. All blood samples were obtained in the morning between 08.00 and 09.00 hours after an overnight fasting, and resting in bed during early follicular phase of the spontaneous or P-induced menstrual cycle. During the same visit, all subjects underwent anthropometric measurements including BMI and detail history, systolic and diastolic blood pressure and Doppler ultrasonographic (US) examination for the evaluation of CIMT. In present study, data regarding the metabolic alteration, oxidative stress markers and CIMT outcomes, and the genetic evaluation of *PROGINS *polymorphism will be shown and discussed.

### Biochemical and hormonal assay

Serum concentrations of hs-CRP were determined by an immunonephelometric assay (N-high-sensitivity CRP; Dade Behring); intra and interassay CV were 1.72 and 2.80%, respectively. Serum total cholesterol, LDL and HDL cholesterol, aspartate aminotransferase (AST), alanine aminotransferase (ALT), and γ-glutamyltransferase (GGT) were measured by Olympus AU 2700 automated analyzer. Plasma insulin concentrations were determined by Immunolite 2000 using two-site chemiluminescent immunometric assay. Serum glucose levels were determined by the glucose oxidase method. Plasma LH, FSH, PRL, oestradiol, progesterone, 17-OHP, testosterone, and dehydroepiandrosterone sulphate were measured by RIA. Insulin resistance was calculated using the homeostasis model assessment insulin resistance index (HOMA-IR) [19], according to the following formula:

HOMA−IR=fasting serum insulin (mU/ml)×fasting plasma glucose (mmol/l)22.5
 MathType@MTEF@5@5@+=feaafiart1ev1aaatCvAUfKttLearuWrP9MDH5MBPbIqV92AaeXatLxBI9gBaebbnrfifHhDYfgasaacH8akY=wiFfYdH8Gipec8Eeeu0xXdbba9frFj0=OqFfea0dXdd9vqai=hGuQ8kuc9pgc9s8qqaq=dirpe0xb9q8qiLsFr0=vr0=vr0dc8meaabaqaciaacaGaaeqabaqabeGadaaakeaacqqGibascqqGpbWtcqqGnbqtcqqGbbqqcqGHsislcqqGjbqscqqGsbGucqGH9aqpfaqabeGabqaabaqbaeaabiqaaaqaaiabbAgaMjabbggaHjabbohaZjabbsha0jabbMgaPjabb6gaUjabbEgaNjabbccaGiabbohaZjabbwgaLjabbkhaYjabbwha1jabb2gaTjabbccaGiabbMgaPjabb6gaUjabbohaZjabbwha1jabbYgaSjabbMgaPjabb6gaUjabbccaGiabcIcaOiabb2gaTjabbwfavjabb+caViabb2gaTjabbYgaSjabcMcaPiabgEna0cqaaiabbAgaMjabbggaHjabbohaZjabbsha0jabbMgaPjabb6gaUjabbEgaNjabbccaGiabbchaWjabbYgaSjabbggaHjabbohaZjabb2gaTjabbggaHjabbccaGiabbEgaNjabbYgaSjabbwha1jabbogaJjabb+gaVjabbohaZjabbwgaLjabbccaGiabcIcaOiabb2gaTjabb2gaTjabb+gaVjabbYgaSjabb+caViabbYgaSjabcMcaPaaaaeaacqaIYaGmcqaIYaGmcqGGUaGlcqaI1aqnaaaaaa@86C9@

### Genetic study

*PROGINS polymorphism *was studied in 95 (genotype success rate 96%) young women with PCOS and 99 healthy women (genotype success rate 94%) control group.

### PROGINS genotyping

#### i-DNA purification

Genomic DNA from patients and healthy controls was extracted from peripheral blood leukocytes using with QIAmp DNA Blood Mini Kits 50 (Qiagen GmbH, Hilden, Germany) according to the manufacturer's instructions. For the PROGINS polymorphism, the following oligonucleotide primers (TIB MOLBIOL Syntheselabor, Berlin-Germany), were used:

Forward **5'TAT GAG CTA TTT GAG TAA AGC CT-3'**

Reverse – 5'-TTC TTG CTA AAT GTC TGT TTT AA-3'

#### ii-PCR conditions

Amplification was carried out on a GeneAmp PCR System 9700(PE Applied Biosystems, Foster City, CA) in a 25 μl reaction mixture in 0.2 ml thin-wall PCR strip tubes (Axygen Scientific, Inc., CA) containing 1 μl genomic DNA solution, GeneAmp Gold Buffer(15 mmol/l Tris-HCl, pH 8.0, 50 mmol/l KCl; PE Applied Biosystems), 1.5 mmol MgCl_2_, 50 μmol/l each of the dGTP, dATp, dTTP and dCTP (Promega, Madison, WI),5 pmol each forward and reverse primers and 1.0 U AmpliTaq Gold polymerase(PE Applied Biosystems). The cycling conditions comprised a hot start at 95°C for 10 min, followed by 35 amplification cycles at 95°C for 30 s, 55°C for 60 s, and 72°C for 45 s, followed by one elongation step at 72°C for 5 min. The PCR products were applied to electrophoretic analysis with the use of a 2% agarose gel Two DNA fragments could be detected: a 185 bp DNA fragment representing 'T1'allele (wild-type) and a 485 bp DNA fragment representing 'T2' allele.

### Methods for plasma MDA, NO, total sulfhydryl group measurements

All reagents were purchased from Sigma and Merck. MDA was determined by a modified spectrophometric method of Yagi K [20] using tetrametoxypropan as Standard and BioTek MicroQuant microplate reader. NO was determined by measuring stable NO end-products-nitrite and nitrate levels using Miranda' s spectrophometric method [21] while total sulfhydryl groups was measured using Ellman's reagent by Sedlak and Lindsay's method [22].

### Radiological measurement

All examinations were performed by one of the two radiologists experienced in US examinations using an equipment of Sonoline Elegra system (Siemens, Erlangen, Germany) with a 7.5 MHz linear-array transducer. The CIMT measurements were performed on the mid portion of the common carotid artery for intima thickness and total thickness including intima and media measurement.

### Statistical analysis

SPSS 14.0 for windows (SPSS Inc. Chicago USA) was used for statistical analysis of the results. P < 0.05 values were accepted as statistically significant. The characteristics of the patients with PCOS and the mean plasma glucose, and insulin, dehydroepiandrosterone sulphate, 17β-estradiol, homocysteine, 17-hydroxyprogesterone, prolactin, testosterone levels between the two clinical groups were compared by Student's t test for unpaired data and between and within the different groups (T1/T1, T1/T2, T2/T2) of *PROGINS *polymorphism with the ANOVA.

Univariate logistic regression analysis was performed in order to show which one of the parameters for which statistically significantly difference was found at Student's t test between patient and control groups was more associated with PCOS. Allelic and genotypic frequencies were determined from observed genotype counts, and the expectations of the Hardy-Weinberg equilibrium were evaluated by χ^2 ^analysis. Differences in the genotype distribution between different groups were assessed by Pearson's χ^2 ^test of heterogeneity. All results were expressed as means ± SD.

## Results

**Figure 1:**
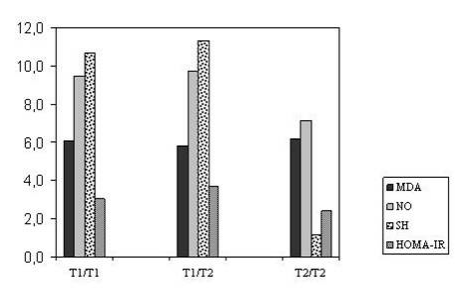
MDA, NO, SH Levels and HOMA-IR Index with PCOS according to The PROGINS genotypes.

Ninety-five PCOS patients and 99 Healthy controls participated in this study. PCOS patients were divided into three sub-groups (T1/T1, T1/T2, T2/T2) according to their PROGINS gene polymorphism. The result of the biochemical, hormonal and oxidative stress parameters and the Carotid intimae media thickness of the PCOS patients sub-groups according to their PROGINS polymorphism is shown in Table 1. In the patients group no statistically significant differences in HDL – cholesterol, LDL – cholesterol, total cholesterol, triglyceride, fasting blood glucose, fasting serum insulin, HOMA-IR, prolactine, f-testosterone, total testosterone, estradiol, DHEAS, fibrinogen, hs-CRP and oxidative stress parameters have been detected (Table 1) (p > 0.05. But a statistically significant difference has been detected on serum FSH levels for T1/T1 genotype according to T2/T2 genotype. In PROGINS polymorphism results; in both control and the patient groups T1/T1 has been detected in high levels (Figure 1). But for genotype (p = 0.178) and allele (p = 0.555) frequencies both of the groups give similar results (Table 2).

**Table 1:** Metabolic and Hormonal Parameters with PCOS According to the PROGINS Genotypes.

	**PCOS Patients (mean ± SD deviation)**			
	T1/T1	T1/T2	T2/T2	P

**Insulin levels (mIU/ml)**	15.3 ± 36.11	15.7 ± 19.01	6.67 ± 4.04	0,39
**Fasting blood glucose (mg/dL)**	90.8 ± 7.7	91.95 ± 11.8	96.67 ± 10	0,55
**s-CRP (mg/dl)**	0.33 ± 0.3	0.657 ± 1.48	0.15 ± 0.05	0,39
**Fibrinogen (mg/dl)**	357.2 ± 88.6	384.2 ± 133	338.6 ± 30.4	0,95
**Right CIMT (milimeter)**	0.42 ± 0.51	0.424 ± 0.43	0.43 ± 0.05	0,85
**Left CIMT (milimeter)**	0.429 ± 0.52	0.424 ± 0.62	0.433 ± 0.57	0,85
**17-OHP (ng/ml)**	5.3 ± 2.8	1.9 ± 0.92	2.2 ± 0.8	0,46
**HDL – Cholesterol (mg/dL)**	56.58 ± 15.3	57.65 ± 14.2	63.6 ± 24.77	0,45
**LDL – Cholesterol (mg/dL)**	117.1 ± 33.1	109.6 ± 27.5	129.0 ± 47.6	0,42
**Triglycerid (mgl/dL)**	110.8 ± 60.1	143.7 ± 88.1	89.3 ± 28.5	0,42
**Cholesterol (mg/dL)**	197.1 ± 44.2	194.0 ± 37.3	210.3 ± 61.7	0,84
**F-testosterone (nmol/Ll)**	2.91 ± 1.6	4.16 ± 2.8	2.5 ± 0.7	0,12
**FSH (mIU/ml)**	5.72 ± 2.16	4.9 ± 1.75	3.23 ± 1.86	0,04

If P < 0.05 in chi-square test it is accepted as statistically significant.

**Table 2:** Genotype and Allele Frequencies of the PRP PROGINS Polymorphism in Women With PCOS And Healthy Controls

**PRPs**	**PCOS Patients (*n *= 95)**	**Control Groups (n = 99)**
**Genotypes**		
**T1/T1**	71 (74.7%)	67 (67.7%)
**T1/T2**	21 (22.1%)	28 (28.3%)
**T2/T2**	3 (3.2%)	4 (3.6%)
**T1/T2 + T2/T2**	24 (25.3%)	32 (31.9%)
**Alleles**		
**T1**	163 (85.8%)	162 (81.8%)
**T2**	27 (14.2%)	36 (18.2%)

*n*, number of patients who could be genotyped successfully.

## Discussion

The importance of this study is working on progesterone receptor polymorphism and searching the effects of this polymorphism on the Oxidative stress markers, inflammatory markers and CIMT of the PCOS patients for whom the gynaecological malignancies show increases. Progesterone and estrogen are the main steroid hormones involved in normal reproduction functions. An abnormality may also occur in PGR receptor except for the progesterone itself. Several polymorphisms have been identified in *PGR*; they include S344T, G393G, V660L, H770H, and the *PROGINS *allele [23,24]. The physiological effects of progesterone are completely dependent on the presence of the human *PGR*, a member of the steroid-receptor superfamily of nuclear receptors [25]. As a consequence of this receptor, PROGINS encodes for a receptor protein with increased stability and increased hormone-induced transcriptional activity [26]. Functional polymorphisms in genes involved in proliferation and cellular homeostasis may affect the risk for benign and malignant disorders.

### Inflammation and progesterone receptor polymorphism (PROGINS)

Chronic inflammation has been associated with a risk of atherosclerosis and cancer development. In previously studies have reported an endothelial dysfunction in women with PCOS [27,28]. Accumulating evidence also suggests that atherosclerosis represents a chronic inflammatory process and inflammatory markers like CRP and fibrinogen, homocysteine provide an adjunctive method for global assessment of cardiovascular risk [29,30].

Recent data also suggest that hs-CRP may directly promote endothelial dysfunction by increasing the synthesis of soluble adhesion molecules, increasing monocyte chemoattractant protein secretion [31,32]. As a result of this study; no difference has been detected on different genotypes of functional progesterone receptor polymorphism (PROGINS) according to hs-CRP and fibrinogen proinflammatory markers, which are important factors for early atherosclerosis in PCOS.

### Insulin resistance and progesterone receptor polymorphism (PROGINS)

The link of PCOS with insulin resistance was subsequently established by clinical studies characterizing the profound insulin resistance in obese and lean PCOS patients. Insulin resistance, hyperinsulinemia, and beta cell dysfunction are very common in PCOS, but are not required for the diagnosis [33].

In PCOS, stimulation of reactive oxygen species (ROS) generation from mononuclear cells (MNCs) by hyperglycaemia may play a role in inflammation through the release of TNFα from circulating MNCs. The oxidative effects of insulin have been demonstrated *in vitro *and in response to both physiological and pharmacological insulin infusions *in vivo *[34,35]. The estradiol hormone level is high in a menstrual cycle, free radicals produced as a consequence of exercise may be easily removed by inactive women with regular menstrual cycles [36]. In this study no statistically significant difference has been detected according to plasma glucose, estradiol level, insulin levels and HOMA-IR between the PCOS patients with different functional progesterone receptor polymorphism (PROGINS).

### Oxidative stress parameters and progesterone receptor polymorphism (PROGINS)

In previously study, did not show a significant correlation between progesterone receptor polymorphism and oxidative system markers in PCOS. It has been reported from experiments in rats that progesterone may also have antioxidant properties in some circumstances [37]. However, these observations have not been reported in humans.

In our study, the Oxidative stress markers have been evaluated in the PCOS patients with progesterone receptor polymorphism (PROGINS). In the presence of inflammation MDA and -SH groups increase but "NO" levels decreases in tissues and blood [38]. Under normal physiological conditions, cellular ROS generation is counterbalanced by the action of antioxidant enzymes and other redox molecules. The balance between O2−
 MathType@MTEF@5@5@+=feaafiart1ev1aaatCvAUfKttLearuWrP9MDH5MBPbIqV92AaeXatLxBI9gBaebbnrfifHhDYfgasaacH8akY=wiFfYdH8Gipec8Eeeu0xXdbba9frFj0=OqFfea0dXdd9vqai=hGuQ8kuc9pgc9s8qqaq=dirpe0xb9q8qiLsFr0=vr0=vr0dc8meaabaqaciaacaGaaeqabaqabeGadaaakeaacqqGpbWtdaqhaaWcbaGaeGOmaidabaGaeyOeI0caaaaa@2FDD@ generation and elimination is important for maintaining proper cellular redox states. A moderate increase in ROS can stimulate cell growth and proliferation [39]. However, excessive ROS accumulation will lead to cellular injury, such as damage to DNA [40], protein, and lipid membrane [41,42]. Because of their potential harmful effects, excessive ROS must be promptly eliminated from the cells by a variety of antioxidant defence mechanisms, including important enzymes, such as superoxide dismutase (SOD), catalase, and various peroxidases. Compelling evidence suggests that cancer cells are generally under ROS stress [43]. Although the precise mechanisms responsible for increased ROS stress in cancer cells have not be defined, the increase in ROS generation is attributed to active cellular metabolic activity under the influence of oncogenic signals and/or to mitochondrial malfunction in cancer cells [44].

In our study no statistically significant difference has been detected according to NO, MDA and -SH levels in the patient groups with different PROGINS genotype. It is known that cancer risk (breast, endometrial) in PCOS patients is higher than the healthy women. Our patients are young and the period that they encountered negative metabolic differences is shorter. Differences on metabolic parameters CIMT and oxidative stress markers for these genotypes may occur after a time period.

In previous studies coronary calcification, as a risk factor of CVD was found to be significantly increased in patients with PCOS in comparison with healthy controls [45,46]. In our study no statistically significant difference has been detected according to f-testosteron, total testosteron and estrogen levels in the patient groups with different PROGINS genotype.

As a result no relation has been detected between the inflammatory and oxidative stress factors, and PROGINS polymorphism alells. This may be because the patients are young and their BMI means are normal and their CIMT and oxidative stress markers are like healthy women. In the future we planned to study on older PCOS patients and evaluate these parameters for this group.

## Abbreviations

PCOS – polycystic ovary syndrome, BMI – body mass index, CI – confidence interval, CV – coefficient(s) of variation, MLRA-Multipl Logistic Regression Analysis, CVD-cardiovascular disease, DHEAS – dehydroepiandrosterone sulphate, E_2_-17β-estradiol, Hcy-homocysteine, 17-OHP-17-hydroxyprogesterone, P – progesterone, PRL – prolactin, T-testosterone, CIMT-Carotid intima-media thickness, BMI-body mass index, FSH-follicle stimulating hormone, LH-luteinising hormone, HOMA-IR – homeostasis model of assessment insulin resistance.
